# 

**Published:** 1887-03-05

**Authors:** 


**The Hospital.**

**An Institution, Family, and Parochial Journal of Hospitals, Asylums, and all Agencies for the Care of the Sick, Criticism and News.**

SATURDAY, MARCH 5TH, 1887.

### THE LITERARY PRIZE.

A PRIZE of **Two Guineas** will be given every month for the best story or incident based upon hospital, or asylum, or student life, or any incident connected with the professions of medicine or nursing.

### HOSPITAL HOUSEKEEPING.

FORMS and full particulars of the Quilter Prizes under this head (see page 355 for 19th Feb.) may be had on application to the Editor of THE HOSPITAL, Norfolk House, W.C. All competitive essays to be sent in by 30th April, 1887. Prizes, £10 10*s.* and £5 5*s.*

### The In- and Out-Patients' Hospital Diary.

This Diary is so arranged as to make some portion of it appear every week, and the whole in each monthly part. It has been very difficult to compile, and corrections will at all times be gratefully received. It has been suggested that similar information about the larger Provincial and Scotch Hospitals would be useful to many readers. We should be glad to hear if this is felt to be the case.

### I.—GENERAL HOSPITALS.

| Hospitals and Terms of Admission. | Physicians, Surgeons, and Medical Officers, with Days and Hours of Attendance. |
|---|---|
| Charing Cross Hospital, West Strand, W.C. Sec., Mr. A. E. Reade<br>Urgent cases are immediately admitted as in-patients. Subscriber's letter expected in other cases<br>Out-patients admitted without subscriber's letter first time; for continued attendance they must be obtained from the Secretary | *In-Physicians*, Drs. Pollock, M. Th.; Green, W. S.; Bruce, Tu. F. Hour, 1.30<br>*In-Surgeons*, Messrs. Barwell, M. Th.; Bellamy, Tu. F.; Bloxam, W. S. Hour, 2<br>*Out-Physicians*, Drs. Wilcocks, M.Th.; Abercrombie, Tu. F.; Lubbock, W. S.; Murray, W. S. Hour, 1.30<br>*Out-Surgeons*, Messrs. Cantlie, M.Th.; J. H. Morgan, Tu. F.; Stanley Boyd, W. S. Hour, 1.30 |
| "Dreadnought" Hospital (Seamen's Hospital Society), Greenwich, S.E. Sec., Mr. W. T. Evans<br>Entirely free to seamen and to all accident cases | *In- and Out-Physicians*, Drs. Curnow, Tu. Th. S.; H. T. Griffiths, M.W.F. Daily, between 9 and 3<br>*In- and Out-Surgeons*, Messrs. W. J. Smith, G. R. Turner. Daily, between 9 and 3 |
| French Hospital, 10, Leicester Place, Leicester Sq., W.C. Sec., Mr. E. Rimmel<br>Free to urgent cases and to all foreigners speaking French | *In-Physician*, Dr. Vintras, Tu. S. 10<br>*In-Surgeons*, Sir W. MacCormac, Tu. 9; Mr. J. Késer, W. F. 10<br>*Out-Physician*, Dr. Vintras, Tu. S. 10<br>*Out-Surgeons*, Messrs. H. de Méric, M. Th. 10; J. Késer, W. F. 10 |
| German Hospital, 113, Dalston Lane, E. Sec., Mr. C. Feldmann<br>Free to natives of Germany, and others speaking German, and to all accidents and emergencies. Others by Governors' letters | *In-Physicians*, Drs. Weber, M. T. 2; Port, Tu. F. 9 a.m.<br>*In-Surgeons*, Drs. Lichtenberg and Burger, W. S. 2<br>*Out-Physicians*, Drs. Tuchmann, Tu. W. 2; Ludwig, M. Th. 2; Keller, Tu. W. F. 2; Philippi, M. Th. F. 2 (*Men*, Tu. Th.; *Women*, M. W. F.) |
| ——— Eastern Branch, 49, Mansell Street, Aldgate, E. | *Out-Physician*, Dr. C. Harrer, Tu. F. 8 to 10 a.m. |
| ——— Western Branch, 259, Oxford Street, W. | *Out-Physician*, Dr. M. Castaneda, M. T. 8 to 10 a.m. |
| Great Northern Central Hospital, Caledonian Road, N. Sec., Mr. W. T. Grant<br>Free to all, without letters of recommendation | *In-Physicians*, Drs. Cholmeley, Cook, and Burnett, M. Tu. W. Th. F. 2<br>*In-Surgeons*, Messrs. W. Adams, W. Spencer Watson, and J. Macready, M. Tu. W. Th. F. 2<br>*Out-Physicians*, Drs. Beale, M. Th. 2; Beevor, Tu. 2, S. 10<br>*Out-Surgeons*, Messrs. Lockwood, M. Th. 2; Makins, Tu. F. 2 |
| Guy's Hospital, St. Thomas's Street, S.E. Supt., Dr. C. J. Steele<br>Patients are admitted, if beds are available, without letters of recommendation, on application at the Hospital. Out-patients are seen free without letters of recommendation | *In-Physicians*, Drs. Pavy, Tu. F.; Pye-Smith, M. Th. S.; Taylor, M. Tu. Th. F. Hour, 1.30<br>*In-Surgeons*, Messrs. Bryant, M. Th. Durham, M. Th. F.; Howse, W. S.; D. Colley, M. Th. Hour, 1.30<br>*Out-Physicians*, Drs. Carrington, W.; Hale White, M.; Goodhart, M. Tu. Th. F. Hour, 12.30<br>*Out-Surgeons*, Messrs. Lucas, Th.; Golding Bird, S.; Jacobson, W.; Symonds, M. Hour, 12.30 |
| King's College Hospital, Portugal Street, W.C. Sec., Mr. T. Mosse Macdonald<br>Accidents and urgent cases free at all times. Other cases by Governors' letters, but where not obtainable, application may be made to the Secretary | *In-Physicians*, Drs. Beale, Duffin, Yeo, Tu. W. F. 1.30; Tu. W. F. S. 2<br>*In-Surgeons*, Mr. Wood, Sir J. Lister, Messrs. H. Smith, Cheyne, W. Rose, Barrow. Daily, 1.30<br>*Out-Physicians*, Drs. Ferrier, J. Curnow, N. I. C. Tirard; *Men*, Tu.Th.; S. 1; *Women*, M. W. F. 1<br>*Out-Surgeons*, Messrs. W. Rose, W. Cheyne; *Men*, Tu. Th. S. 1; *Women*, M. W. F. 1 |
| London Hospital, Whitechapel Road, E. Sec., Mr. A. Haggard<br>Accidents and urgent cases free; out-patients for dental and maternity departments need no recommendation; other cases by subscriber's order. Out-patients are required to attend only once, unless otherwise directed by the Physician or Surgeon | *In-Physicians*, Drs. Mackenzie, Tu. F.; Down, Tu. F.; H. Jackson, M. Th.; Sutton, M. Th.; Fenwick, Tu. F. 1.30<br>*In-Surgeons*, Messrs. Rivington, Tu. F.; Couper, Waren Tay, M. Th.; McCarthy, W.S.; Treves, Tu.F. Hour, 1.30<br>*Out-Physicians*, Drs. Sansom, Th.; M. Ralfe, M. Th.; Turner, Tu. F.; Warner, Tu. F.; Gilbert Smith, W. S. J. Anderson, W. S. Hour, 1.30<br>*Out-Surgeons*, Messrs. Mansell-Moullin, M. Th.; Eve, M. W.; Reeves, Tu. S.; H. Fenwick, Tu.F. Hour, 1.30 |

| Hospitals and Terms of Admission. | Physicians, Surgeons, and Medical Officers, with Days and Hours of Attendance. |
|---|---|
| London Temperance Hospital, Hampstead Road, N.W.<br>For treatment without alcohol. By letter or small payment | *In- and Out-Physicians*, Drs. J. Edmunds, M.; J. J. Ridge, Tu. F. Hour, 1.30<br>*In- and Out-Surgeons*, Mr. A. P. Gould, Th. 2.30 |
| Metropolitan Free Hospital, Kingsland Road, N. Sec., Mr. G. Croxton<br>Admission free to the sick poor, without any letter of recommendation. Urgent cases at all times | *In- and Out-Physicians*, Drs. Drysdale, Tu. F. 12; J. G. Dudley, W. S. 12; Fotherby, M. Th. 12; H. H. Tooth, Tu. F. 9; A. Haig, W. S. 9; A. Davies, M. Th. 9<br>*In- and Out-Surgeons*, Messrs. E. J. Chance, W. S. 9; Goodsall, Tu. F. 9; Walsham, M. Th. 9; A. A. Bowlby, F. 9; S. Paget, Th. 9; C. J. Thompson, daily, 9 |
| Middlesex Hospital, Berners Street, W. Sec. and Supt., Mr. A. O. D. Bartholeyns<br>Urgent cases admitted at all times; other cases by ticket. All medical cases must, in the first instance, be seen by the Resident Medical Officer, who attends at the hospital daily, at 11 a.m. | *In-Physicians*, Drs. Cayley, M. Th.; Coupland, Tu. F.; D. Powell, M.Th.; Finlay, Tu. F. Hour, 1.30<br>*In-Surgeons*, Messrs. Hulke, M. Th.; Lawson, M. Th.; Morris, Tu. F. Hour, 1.30<br>*Out-Physicians*, Drs. Fowler, Tu. F. 3.30; Biss, M. Th. 1.30; Pringle, W. S. 1.30<br>*Out-Surgeons*, Messrs. Andrew Clark, M. 1.15 (*Men*); Th. 1.15 (*Women*); Pearce Gould, F. 1.30 (*Men*); Tu. 1.30 (*Women*); J. B. Sutton, W. S. 9 |
| Miller Hospital and Kent Dispensary, Greenwich, S.E. Hon. Sec. Mr. W. Bristow. | *Physicians*, Drs. T. Creed, C. H. Hartt, G. Willes<br>*Surgeons*, Messrs. T. Moore, J. Poland, E. Clarke |
| North-West London Hospital, 18, Kentish Town Road, N.W. Sec., Mr. A. Craske<br>By subscribers' tickets or payment, but accidents and urgent cases free at all times | *In- and Out-Physicians*, Drs. W. C. Hood, J. Shaw, Edwarde H. Campbell. Daily, 1.30<br>*In- and Out-Surgeons*, Messrs. F. Durham, Collier, Jennings, J. Black. Daily, 1.30 |
| Ospedale Italiano, 41, Queen Square, Bloomsbury. Sec., Sig. L. Bonacina<br>Free to all, but Italians have preference | *In- and Out-Physicians*, Drs. M. Castaneda, Tu. F. 10 to 11; B. Sassella, M.Th. 10 to 11; Mr. J.W.B. Steggall, W.S. 4 to 5 |
| Poplar Hospital, 303, East India Dock Road, E. Sec., Lt.-Col. Feneran<br>Free to accidents and emergencies | *In-Surgeons*, Messrs. F. M. Corner, M. Brownfield, and Resident Surgeons<br>*Out-Surgeons*, Dr. J. D. Grant, W. S. Mr. T. E. Bowkett, Tu. F. 12 to 2 |
| Royal Free Hospital, Gray's Inn Road, W.C. Sec., Mr. J. S. Blyth<br>Admission free to the sick poor, without any letter of recommendation | *In- and Out-Physicians*, Drs. Cockle, M. Th.; Sainsbury, Tu. F.; West, W. S. Hour, 1<br>*In- and Out-Surgeons*, Messrs. Rose, M. Th.; W. Gant, Tu. F.; Barrow, W.S. Hour, 1 |
| St. Bartholomew's Hospital, Smithfield. Clerk, Mr. W. Cross<br>Admission free, without letter or ticket. Accidents and other urgent cases admitted at any time; ordinary cases of disease daily, between 9 and 10. Patients are seen for Electrical treatment on M. Tu. Th. and F. 1.30 | *In-Physicians*, Drs. Andrew, Church, Gee, Sir D. Duckworth. Daily, 1.30<br>*In-Surgeons*, Messrs. Savory, T. Smith, Willett, Langton, M. Baker. Daily, 1.30<br>*Out-Physicians*, Drs. Hensley, M. Th. 11; Brunton, Tu. F. 11; Legg, W. S. 11; N. Moore, Tu. W. Th. S. 9<br>*Out-Surgeons*, Messrs. Marsh, Tu. F.; 12; Butlin, M. Th. 12; Walsham, W. S. 12; Cripps and Bruce Clarke, daily, 9<br>*Casualty Physicians*, Drs. Davies, Tu. W. F. S.; O. Browne, M. Tu. Th. F.; Nias, M. W. Th. S. Hour, 9 |
| St. George's Hospital, Hyde Park Corner, S.W. Sec., Mr. C. L. Todd<br>Free to accidents and emergencies. Other in-cases by Governor's or subscriber's letter, but, in necessitous cases, this recommendation is not strictly insisted on. Letters are not required for out-patients. Vaccination on Th. at 11 | *In-Physicians*, Drs. Wadham, M. F.; Dickinson, M. Th. Tu. F.; Whipham, Tu.W. S.; Cavafy, Tu. S. Hour, 1<br>*In-Surgeons*, Messrs. Holmes, M. Th. F. 1, W. 1.30; Rouse, M. Th. F. 1, W. 1.30; Haward, Tu. Th. S. 1, W. 1.30<br>*Out-Physicians*, Drs. Ewart, M. F.; Owen, Tu. S. Hours, 10.30 to 12<br>*Out-Surgeons*, Messrs. Bennett, M. F.; Dent, Tu. S. Hours, 10.30 to 12 *Men*, F. S.; *Women*, M. Tu. |

### THE CHILDREN'S CORNER.

### OUR PASTIMES.

**Children's Quarterly Prize Competition.**
Began 1st Jan., 1887 ; ends 26th Mar., 1887.

PRIZES.
FOR MOST CORRECT ANSWERS.

| | | | |
|---|---|---|---|
| 1st .. | .. Thirty Shillings | 3rd .. | .. Ten Shillings |
| 2nd .. | .. Twenty „ | 4th .. | .. Five „ |

FOR NEATEST ANSWERS.
Five Shillings.
FOR BEST LITTLE LETTERS.
Ten Shillings.

**Tenth Competition.**

No. 52. A PUZZLE IN SUBTRACTION.
From nineteen take the fourth of four,
And leave remainder just an even score.

No. 53. CONUNDRUM. Why was Noah the greatest financier of his time?

No. 54. FLOWERS ENIGMATICALLY EXPRESSED. A cunning animal and a part of our dress; a colour and a carillon; something we eat on bread, and something we drink out of. Tell me, please, the names of the three flowers.

No. 55. ARITHMETICAL PUZZLE. Convert 100+1+5+1+50 into a word which means polite.

No. 56. HIDDEN CHRISTIAN NAMES. "Agamemnon in a haste desired to sail from Aulis. A calm arose, as the anger of Diana, sweet omnipotent goddess, allured and kept him. He had one daughter, Iphigenia, daring and brave. To her nestling in his arms he says, 'Iphigenia, my daughter, I called thee; verily Calchas, famed for prophetic art, hurries thee to death, already in thy youth, and bids thy sacrifice.'" I want you to discover in the above, thirteen Christian names. The proper names already there do not count.

**Results of Eighth Competition.**

No. 48. CONUNDRUM. That which is as white as chalk, as light as down, as soft as silk, as moist as froth, is Snow.

No. 44. GEOGRAPHICAL ENIGMA. The river is the Amazon.

No. 45. HOUR-GLASS PUZZLE:—Nurse.

p e N c e d U e

R

a S k f l E e t

No. 46. BURIED TREES. Ash, Oak, Elm, Lime, Pine, Larch.

*All right*—Mikado, Dot, Joe, Scrooge, P. S., Ellie, Florrie, Skye, Tim, Peeler, A. C. K.; *Three right*—Paul Methven, Prospect, Alsie, Little Mary, Ariadne; *Two right*—Sutton.

ERRATUM.—I am sorry that, through inadvertence, an error was made in the answer of No. 42. It should have been "His son's." Credit for correct answers is accordingly given to Tim, Mikado, Scrooge, A. C. K., Peeler, Skye, Ellie, Prospect, Joe, and Dot.

**Letters from Little Folk.**

My weekly budget of correspondence grows not only in interest but also in quality. Next week the subject of composition will be "Amusements." Tell me something of the games you like best. Here are one or two letters which I have received about "Books," but I have to "scold" nearly all my little friends for having overlooked the new rule requiring *the age of the competitors to be appended to their signatures.*

DEAR MR. EDITOR.—As you have asked us to write about books, I am very pleased to have the opportunity of telling you that I obtained my first prize, *The Wide, Wide World* for regular attendance at the Stockwell Road Board School in November last, and liked it very much. In fact, I was so delighted with the character of Ellie, that I have chosen her name as my *nom de plume*.—Yours faithfully, ELLIE, aged 9.

44, Burgoyne Road, Brixton.

DEAR MR. EDITOR,—I like you to tell us something to write about. I am very fond of reading, and have many favourite books. One is called *Holiday House.* It is about a boy called Harry and his sister Laura. They were always in mischief, and as they had a very cross nurse, called Mrs. Crabtree, they were always in trouble. Once Laura cut off all her beautiful long curls, and at the same time Harry set the night nursery on fire, but they were so sorry afterwards that their grandmamma soon forgave them. Once, when their grandmamma was away from home, they sent out notes, and invited quite a large party of little friends to "a grand party," and old Peter laid the table so nicely for them, but Mrs. Crabtree would only let them have the two cups of milk, and two hard, dry biscuits that Harry and Laura always had for their supper, so all the little children had to go home without tea or supper; but when their grandmamma heard all about it, she invited them all over again, and gave them a splendid feast, but they never forgot Harry's "grand feast."

Yours truly, MIKADO, aged 13.

12, Upper Tichborne Street, Leicester.

**Letter Prize.**

One mark each: Mikado, Scrooge, P. S., Ellie, Florrie, Skye, Tim, Peeler, Halford.

**Notices to Correspondents.**

HALFORD.—I know the first book you mention, *Adventures in India*; it is exceedingly full of interest.

PEELER.—I, too, have admired the character of Nelly in the *Old Curiosity Shop.* I have always regarded it as one of Dickens's happiest conceptions.

FLORRIE.—No one can fail to like *David Copperfield.* Dickens's depiction of character, especially in the lower phases of life, is simply marvellous.

Answers must be addressed to the "PUZZLE EDITOR," THE HOSPITAL, Norfolk House, Norfolk Street, Strand, W.C., not later than Thursday next. The "Children's Corner" Coupon (see advertisement pages) must be enclosed.

### AMUSEMENTS WITH PROFIT.

### PRIZE COMPETITIONS.

**Quarterly Prize Competition.**
Began 22nd January ; Ends 16th April.

**A Prize of Three Guineas**
will be awarded to the Competitor who shall have sent in the largest number of correct or best answers. No one will be permitted to win the Three Guinea Prize twice in any one year, but we shall award annually an additional
**Prize of Five Guineas**
to the competitor who shall have sent in the largest number of correct or best answers during the twelve months.

**No. 13. Double Acrostic.**

*Primals*—A town for its *cutlery* noted
*Finals*—A place to the *sick poor* devoted.

'Tis yet remembered as Jebel Mûsa's *height*
Where the Law was given to the Israelite.

Either a noun or an adjective is that
Relating to *Erin*, the land of poor Pat.

A *creature of fable*, a puissant king of night,
An imp or a fay, a goblin or a sprite.

In old Rome these *registers* of varied kind
Gave fête days, courts, and the consuls' names enshrined.

In Scotia a *county*, traversed by Strathmore,
Which in Keltic days the name of Mormaer bore.

'Mong the Ulema, in the Moslem's belief,
'Tis an honour'd *teacher*; the Sultan is chief.

Lo, a *mighty mountain*, where Vulcanus wrought
At least, in mythology, so we are taught.

A *monk*, a Titan in intellectual strength,
Whose grand work shook Christendom throughout its length.

A charming old *diarist* whose versatile pen
Recorded whatsoe'er came under his ken.

**No. 14. Charade.**

Just a cypher, write it down;
To this you'll please annex a ten;
Add to this three-fourths of four;
To this append five hundred, then.
These few numbers set aright
Disclose a city of great fame,
It is for its *letters* known,
Though *figures* will spell out its name.

**Answers.**

No. 9. DOUBLE ACROSTIC.

H ero D

O port O

S u N

P anam A

I ngo T

T ermin I

A jacci O

L io N

S poke S

Correct answers have been received from A. M. E., Robe, Gretta, Paddy, Ostrich, Miss Larkins, C. le Maître, K. M., Condorcet, Mr. J. High, Gip, Mater, Patient, Ellice, H. B., Peeler, H. J. C., Hexameter, Mrs. Bedingfeld, Perseverance.

NO 10. ARITHMETICAL QUESTION.

From the conditions, it is clear that the woman must have sold to the third guard 37 eggs; to the second, 74; and to the first, 148. As she had 36 eggs remaining when she arrived at the market place, she must have started with 295.

Correct answers have been received from Robe, Gretta, Paddy, Ostrich, C. le Maître, K. M., Condorcet, Mr. J. High, Gip, Mater, Patient, Ellice, Peeler, H. J. C., Hexameter.

Answers must be addressed to THE PRIZE EDITOR, AMUSEMENTS WITH PROFIT, THE HOSPITAL, Norfolk House, Norfolk Street, Strand, W.C., not later than Saturday next. The full name and address must in all cases be given, but a *nom de plume* may be added for publication. Only one side of the paper should be written on. The "Amusements with Profit" Coupon (see advertisement pages) must be enclosed with replies.

